# Population Normative Data for Commonly Used Neuropsychological Measures in Older Nigerian Adults in the READD‐ ADSP study

**DOI:** 10.1002/alz70857_106700

**Published:** 2025-12-25

**Authors:** Andrew F Zaman, Pedro R Mena, Reginald Obiako, Katalina F. McInerney, Njideka U Okubadejo, Larry D Adams, Olufisayo Oluyinka Elugbadebo, Goldie S Byrd, Allison M Caban‐Holt, Christiane Reitz, Oluwadamilola Ojo, Giuseppe Tosto, Paula K. Ogrocki, Brian W Kunkle, Jonathan L Haines, Jeffery M Vance, Olusegun Baiyewu, Margaret Pericak‐Vance, Adesola Ogunniyi, Stella‐Maria Paddick, Michael L Cuccaro, Rufus O. Akinyemi

**Affiliations:** ^1^ University of Miami Miller School of Medicine, Miami, FL, USA; ^2^ John P. Hussman Institute for Human Genomics, University of Miami Miller School of Medicine, Miami, FL, USA; ^3^ Ahmadu Bello University, Zaria, Kaduna, Nigeria; ^4^ Department of Neurology, University of Miami Miller School of Medicine, Miami, FL, USA; ^5^ College of Medicine, University of Lagos, Lagos, Nigeria; ^6^ University College Hospital, Ibadan, Oyo, Nigeria; ^7^ Wake Forest University School of Medicine, Winston‐Salem, NC, USA; ^8^ Gertrude H. Sergievsky Center, Taub Institute for Research on the Aging Brain, Departments of Neurology, Psychiatry, and Epidemiology, College of Physicians and Surgeons, Columbia University, New York, NY, USA; ^9^ College of Medicine, University of Lagos, Lagos, Lagos, Nigeria; ^10^ Department of Neurology, Case Western Reserve University School of Medicine, Cleveland, OH, USA; ^11^ Department of Neurology, University Hospitals Cleveland Medical Center, Cleveland, OH, USA; ^12^ Dr. John T. Macdonald Foundation Department of Human Genetics, University of Miami Miller School of Medicine, Miami, FL, USA; ^13^ Cleveland Institute for Computational Biology, Case Western Reserve University, Cleveland, OH, USA; ^14^ Department of Population & Quantitative Health Sciences, School of Medicine, Case Western Reserve University, Cleveland, OH, USA; ^15^ College of Medicine, University of Ibadan, Ibadan, Nigeria; ^16^ College of Medicine, University of Ibadan, Ibadan, Oyo, Nigeria; ^17^ Population Health Institute, Newcastle University, Newcastle, United Kingdom; ^18^ College of Medicine, University of Ibadan, Ibadan, Oyo State, Nigeria; ^19^ Institute of Advanced Medical Research and Training, College of Medicine, University of Ibadan, Ibadan, Oyo State, Nigeria

## Abstract

**Background:**

Diagnostic classification of neurocognitive disorders (e.g., Alzheimer's disease) relies heavily on norm‐based neuropsychological tests. Classification is optimized by the use of population‐specific norms to provide appropriate reference groups. For this study, we established key adjustments for commonly used US and African neuropsychological measures in the Recruitment and Retention for Alzheimer's Disease Diversity Genetic Cohorts in the Alzheimer's Disease Sequencing Project (READD‐ADSP).

**Method:**

Our dataset was drawn from older adults residing in three urban sites in Nigeria (Ibadan, Lagos, and Zaria) who were enrolled in the READD‐ADSP study. Eligible participants were adjudicated as non‐cognitively impaired; had no impairment on the Clinical Dementia Rating (CDR); a score of >=7 on the Intervention for Dementia in Elderly Africans (IDEA) cognitive screen; no functional impairment; no neuropsychiatric concerns; and no major health concerns/diseases. The dataset consisted of 226 individuals (71.3±6.7 years old, 43% Male, 8.9±6.3 years of education, and 73% were literate). Linear regression analyses estimated the effect of age, sex, education, and literacy (yes/no) on select neuropsychological tests including: Montreal Cognitive Assessment (MoCA), CERAD Word List, Category Fluency (Animal Naming), Verbal Fluency, Multilingual Naming Test (MINT), Number Span, Stick Design (SDT), and IDEA Cognitive screen (IDEA).

**Result:**

R^2^ values for the 12 regression models ranged between 0.32 (Verbal Fluency) and 0.05 (Stick Design Immediate); nine of the models were significant; three models failed to attain significance (CERAD (Immediate and Delay) and the IDEA. Years of education was the most common significant factor. Individuals with greater education performed better on the MoCA, Animal Naming, Verbal Fluency, MINT, Numbers Forward, and Numbers Backward. Literacy was significant for three tests (Vegetable Naming, Numbers Forward, and SDT‐Delay. Age was significant for Animal Naming, MINT, SDT (Immediate and Delay). Finally, sex was significant for CERAD (Immediate, Vegetable Naming, and Numbers Backwards.

**Conclusion:**

This study is a first step in addressing the need to develop test norms in the Nigeria population. Not surprisingly, years of education was often the most robust factor that needed to be adjusted for in these countries. We can use the regressions/normative values to provide age, sex, education, and literacy adjusted Z‐scores to clinical adjudicators.

